# Effect of Vitamin D Status on Vascular Function of the Aorta in a Rat Model of PCOS

**DOI:** 10.1155/2021/8865979

**Published:** 2021-03-18

**Authors:** K. Lajtai, R. Tarszabó, B. Bányai, B. Péterffy, D. Gerszi, É. Ruisanchez, R. E. Sziva, Á. Korsós-Novák, R. Benkő, L. Hadjadj, Z. Benyó, E. M. Horváth, G. Masszi, S. Várbíró

**Affiliations:** ^1^Department of Obstetrics and Gynecology, Semmelweis University, Hungary Budapest, Üllői út 78/a, H-1082; ^2^Department of Obstetrics and Gynecology, Markusovszky Lajos University Teaching Hospital, Hungary Szombathely, Markusovszky Lajos utca 5., H-9700; ^3^Department of Physiology, Hungary Budapest, Tűzoltó utca 37-47, H-1094; ^4^Department of Translational Medicine, Hungary Budapest, Tűzoltó utca 37-47, H-1094; ^5^Department of Pathology, Hetényi Géza Hospital, Hungary Szolnok, Tószegi út 21, H-5000; ^6^Department of Cardiology, Bajcsy-Zsilinszky Hospital, Hungary Budapest, Maglódi út 89-91, H-1106

## Abstract

Polycystic ovary syndrome (PCOS) is associated with elevated cardiovascular risk. Early vascular dysfunction may lead to the development of cardiovascular disease in PCOS. Vitamin D deficiency (VDD) is a common comorbidity of PCOS that contributes to the pathogenesis of the disease and its complications. Both PCOS and VDD are accompanied by increased oxidative stress that may be involved in the arising vascular dysfunction. We aimed to investigate the role of vitamin D status on aortic function. PCOS was induced by an 8-week-long transdermal testosterone treatment of female rats, and low and adequate vitamin D status was achieved by dietary means. Contraction and relaxation abilities of isolated aortic segments were measured by myograph. Resorcin-fuchsin staining and immunohistochemical labeling of 3-nitrotyrosine were performed. No difference was shown in the norepinephrine-induced contraction of the aortas of different groups, whereas we detected reduced acetylcholine- and insulin-evoked relaxation in VDD groups. A lower level of resorcin-fuchsin staining and elevated 3-nitrotyrosine immunostaining was observed in VDD. In our study, we demonstrated early endothelial dysfunction in VDD PCOS rat model. Vitamin D supplementation could prevent vascular disturbances, while VDD itself damaged endothelium-dependent vasorelaxation and induced nitrative stress.

## 1. Introduction

Polycystic ovary syndrome (PCOS) is a common endocrine disorder affecting 15-20% of fertile age women worldwide [1]. Diagnosis is based on the Rotterdam criteria, which requires two of the following three conditions: oligo-anovulation, hyperandrogenism, or polycystic ovary morphology [2]. Most often, patients tend to seek medical attention due to fertility-related problems, menstrual cycle disorders, or hirsutism. However, long-term health risks seem to have an even greater impact on the quality of their life: PCOS is associated with a variety of subsequent comorbidities including insulin resistance, type 2 diabetes mellitus, and an increased cardiovascular (CV) risk. PCOS women have a higher probability of developing hypertension and coronary artery disease in later life. Endothelial dysfunction is an early predictor of emerging future CV morbidities. In young PCOS patients, reduced brachial artery flow-mediated dilation has been detected as a sign of compromised endothelial function [3].

Several underlying mechanisms have been identified, ranging from vitamin D deficiency (VDD) to oxidative stress. VDD can be identified in 70-80% of PCOS women [4, 5]. VDD itself is associated with various morbidities including metabolic syndrome, obesity, and cardiovascular disease. It has been hypothesized that treating VDD can ameliorate PCOS as in some studies, and vitamin D treatment succeeded to increase ovulation rates [6].

Vitamin D binding to VDR (vitamin D receptor) has effect in a genomic as well as a nongenomic manner. It is able to modify target gene expression and intracellular signal transduction. Vitamin D has a well-defined antioxidant activity—subsequently, VDD induces oxidative stress and endothelial dysfunction. Vitamin D suppresses the gene expression of nuclear factor-*κ*B (NF-*κ*B) and tumor necrosis factor- *α* receptor 2 and 4. NF-*κ*B is a transcription factor responsible for increasing the production of proinflammatory factors such as advanced glycation end products (AGEs), interleukin 1 and 6 (IL-1 and 6), TNF-*α*, and monocyte chemoattractant protein 1 (MCP-1). Furthermore, TNF-*α* upregulates c-Jun-N-terminal kinase (JNK). JNK inhibits eNOS and induces xanthine oxidase (XO), which is responsible for elevated superoxide production. These two mechanisms both lead to a reduced NO bioavailability and consequently, an emerging endothelial dysfunction [7].

Our previous research using a DHT-induced rat model of PCOS focused on detecting vascular dysfunction in various vessels of female rats and the positive effect of vitamin D supplementation. However, the possible deteriorative effect of VDD has not been investigated in similar models yet.

In the present study, we developed a combined (hyperandrogenic and vitamin D deficient) PCOS model to further examine the effect of VDD in hyperandrogenic milieu, as well as in normal female endocrine status. The setup enabled us to examine the isolated effect of hyperandrogenism and VDD on the aorta's vascular function. We aimed to analyze the early changes in vascular function and nitrative stress without later complications such as hypertension or type 2 diabetes.

## 2. Methods

### 2.1. Chemicals

Aortic function was measured ex vivo in Krebs-Ringer (KR) solution (in mmol/l): NaCl 119, KCl 4.7, NaH_2_PO_4_ 1.2, MgSO_4_ 1.17, NaHCO_3_ 24, CaCl_2_ 2.5, glucose 5.5, and EDTA 0.034. The solution was freshly prepared each day; the temperature was kept at 37°C. Stable pH was achieved by bubbling with a gas mixture (containing O_2_ 20%, CO_2_ 5%, and N_2_ 75%). Norepinephrine (NE) and acetylcholine (Ach) were purchased from Sigma-Aldrich (St Louis, MO, USA). Human-recombinant insulin (Actrapid pentafill 100 IU/mL) from Novo Nordisk (Bagsvaerd, Denmark) was used for in vitro vascular tests.

### 2.2. Animals

The study was performed in accordance with the Guide for the Care and Use of Laboratory Animals published by the US National Institutes of Health (8th edition, 2011) and the EU conform Hungarian Law on Animal Care (XXVIII/1998). The institutional Animal Care Commission and Hungarian authorities have confirmed the research protocol (IRB: 8/2014, PEI/001/1548-3/2014).

48 adolescent (21-28 day-old), 100-140 gram-weighing female Wistar rats were purchased from Charles River (Charles River Ltd., AnimaLab, Vác, Hungary) and kept at the Animal Facility of Semmelweis University. Random assignment was carried out forming four experimental groups as follows: hyperandrogenic vitamin D deficient group (T + D-, *N* = 12), hyperandrogenic vitamin D supplemented group (T + D+, *N* = 12), vitamin D deficient group (T-D-, *N* = 12), and vitamin D supplemented group (T-D+, *N* = 12).

### 2.3. Chronic Treatment of the Rats

The hyperandrogenic state was induced by an 8-week-long transdermal testosterone treatment. 0,0333 mg/g of Androgel (50 mg/5 ml gel by Lab. Besins International S.A.) was applied 5 times a week on a previously shaved 3 × 3 cm area on the back of the animals.

Vitamin D intake was reduced to generate vitamin D deficiency with the animals being fed with Vitamin D Free Lab Rat/Mouse Chow (ssniff Spezialdiäten GmbH, Soest, Germany) containing less than 5 IU/kg vitamin D3. Vitamin D supplemented rats were kept on a regular chow containing 1000 IU/kg of vitamin D. Furthermore, additional oral vitamin D supplementation was organized as the following: 500 IU cholecalciferol on the second week and a weekly dose of 140 IU/100 g on the fifth, sixth, and seventh weeks (Vigantol (cholecalciferol) 20000 IU/ml, Merck/Merck Serono, Darmstadt, Germany) were administered using a gavage cannula. Dosage was calculated based on human vitamin D supplementation guidelines that suggest an ideal serum 25-OH-cholecalciferol level of 25-50 ng/ml [8, 9]. The animals were provided with the appropriate amount of rat chow and tap water. Rats were housed at constant room temperature (22°C ± 1°C) in 12 h/12 h light-dark cycle. Two rats were placed in each cage.

After the 8-week-long treatment, Nembutal anaesthesia (45 mg/kg intraperitoneal) was performed. The cardiovascular system was infused with heparinized KR for 2 minutes before isolating the thoracic aorta. The thoracic aortic segment was isolated and cut into 9 equal pieces, each approximately 3 mm long. 8 of these were used for functional testing on a conventional wire myograph setup (610-M MultiMyograph System; Danish Myo Technology A/S, Hinnerup, Denmark). The remaining aortic ring was fixed in formalin and embedded in paraffin.

### 2.4. Myography

Isometric tension of isolated thoracic aortic rings was measured via wire myography. Organ chambers were filled with 8 ml KR solution. A constant temperature was set at 37°C. 15 mN pretension was reached in a progressive fashion. Having achieved stable pretension, 124 mmol/L K^+^ was administered (3 min) to verify the vessels' contractile ability and provide a setpoint of maximum value for contraction force. Then, K^+^ was washed out with KR solution, and the contraction force after cumulative concentrations of NE (10^−9^, 10^−8^, 10^−7^, and 10^−6^ mol/L) was measured. After maximal contraction, we examined vasorelaxant potential using Ach in increasing concentrations (10^−8^, 10^−7^, 10^−6^, and 10^−5^ mol/L). After another equilibration with KR, precontraction was newly generated by norepinephrine (5 × 10^−8^ mol/L). Insulin-mediated relaxation capacity was examined at four cumulative concentrations (60, 120, 300, and 600 IU/L).

### 2.5. Histology

Paraffin-embedded tissue sections received a classical resorcin-fuchsin (RF) stain (Sigma).

Immunohistochemistry was performed on paraffin-embedded tissue sections of the thoracic aorta against 3-nitrotyrosine (NT). After deparaffinization, antigens were retrieved by heating the slides in citrate buffer (pH = 3). We blocked endogenous peroxidase activity with 3% H_2_O_2_ in dH_2_O_2_. Nonspecific labeling was evitable using 2.5% normal horse serum (Vector Biolabs, Burlingame, CA, USA). After overnight application of primary antibodies (polyclonal rabbit anti-NT 1 : 500, Merck Millipore, Burlington, MA, USA) at 4°C, horseradish-peroxidase- (HRP-) linked anti-mouse monoclonal horse antibodies (Vector Biolabs) provided secondary labeling which was visualized by brown-colored diamino-benzidine (DAB, Vector Biolabs). For counterstaining, blue-colored hematoxylin (Vector Biolabs) was utilized. Zeiss Axio Imager system (Zeiss, Oberkochen, Germany) was used for microscopic imaging of tissue sections. Uncalibrated optical density of brown coloring was estimated by ImageJ software (NIH, Bethesda, MA, USA).

### 2.6. Statistics

Effects of testosterone treatment and vitamin D status were evaluated by nonparametric Kruskal-Wallis test with Dunn's multiple comparison by Prism 8 (GraphPad, GraphPad Software, USA). Vascular function curves were analyzed by repeated-measures two-way ANOVA using Bonferroni's post hoc test. *p* < 0.05 was uniformly accepted as the threshold for statistical significance.

## 3. Results

### 3.1. Vascular Function

**Figure 1:**
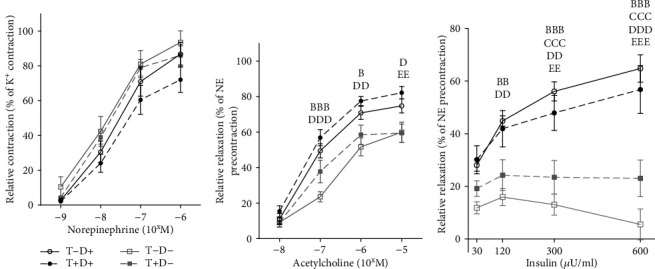
(a) Relative contractile ability of the aorta in female rats after induced by cumulative concentrations of NE. Contraction was calculated as the percentage of maximal contraction reached after a previous 3-minute K^+^ treatment (124 mmol/L). There was no difference in the developed vasoconstriction among treatment groups regardless of norepinephrine concentration. *N* = 10 − 11 in each group. Data are presented as mean ± SEM. (b) Acetylcholine-mediated vasorelaxation. Dilation was compared to precontraction induced by 5 × 10^−8^ M norepinephrine. Significantly reduced relaxation was measured in VDD groups, starting at 10^−7^ ACh concentration, remaining significant in growing concentrations. *N* = 9 − 11 in each group. Two-way ANOVA, Tukey's post hoc test. bbb: T-D- vs. T-D+ *p* < 0.001, b: T-D- vs. T-D+ *p* < 0.05, ddd: T-D- vs. T + D+ *p* < 0.001, dd: T-D- vs. T + D+ *p* < 0.01, d: T-D- vs. T + D+ *p* < 0.05, ee: T + D- vs. T + D+ *p* < 0.01. Data are presented as mean ± SEM. (c) Insulin-dependent vasodilation. Relaxation was calculated in comparison to precontraction induced by 5 × 10^−8^ M NE administration. Vitamin D deficient groups had compromised insulin-dependent vasodilatation in 120 mikroU/ml insulin concentration and above. *N* = 8 − 10 in each group.Two-way ANOVA, Tukey's post hoc test. bbb: T-D- vs. T-D+ *p* < 0.001, bb: T-D- vs. T-D+ *p* < 0.01, ccc: T + D- vs. T-D+ *p* < 0.001, ddd: T-D- vs. T + D+ *p* < 0.001, dd: T-D- vs. T + D+ *p* < 0.01, d: T-D- vs. T + D+ *p* < 0.05, eee: T + D- vs. T + D+ *p* < 0.001, ee: T + D- vs. T + D+ *p* < 0.01. Data are presented as mean ± SEM.

The contraction ability of the aortae was maintained in all experimental groups shown by intact NE-induced vasoconstriction of the isolated vascular segments (Figure 1(a)). This suggests a well-preserved smooth muscle cell function of the aortic wall. On the other hand, an impaired endothelial function could already be detected as reduced Ach-mediated vasorelaxation was measured in the vitamin D deficient groups. T-D- group showed significantly diminished dilatation compared to both D+ groups. The difference gained significance at 10^−7^ M Ach concentration and remained so in greater concentrations. At 10^−5^ M, the T + D- group was also significantly less relaxed compared to T + D+ (Figure 1(b)).

Similarly to the emerging systemic insulin resistance [10] observed in VDD treatment groups, compromised insulin-dependent vasodilation was registered. Significantly reduced relaxation was measured at 120 IU/L insulin concentration and above. (Figure 1(c)).

### 3.2. Histology

**Figure 2:**
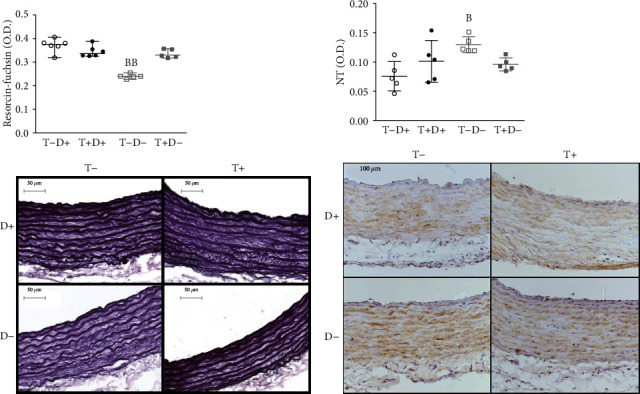
(a) Resorcin-fuchsin staining in aortic tissue. Conventional resorcin-fuchsin staining was applied to aortic rings then noncalibrated optical density was measured. A significantly lower staining density was detected in T-D- group compared to the control. *N* = 5 − 6 in each group, Kruskal-Wallis test, Dunn's multiple comparisons test. bb: T-D- vs. T-D+ *p* < 0.01. Data are presented as individual data points; lines represent median [IQR]. (b) Immunohistochemical measurement of 3-nitrotyrosine (NT) staining intensity in the aortae of female rats. NT immunostaining is visualized by brown-colored DAB, while hematoxylin is used as counterstaining. We detected significantly elevated staining density in T-D- rats compared to controls. *N* = 4 − 7 in each group, Kruskal- Wallis test, Dunn's multiple comparisons test. b: T-D- vs. T-D+ *p* < 0.05. Data are presented as individual data points; lines represent median [IQR].

Analysis of histologic evaluation of aortic tissue sections revealed significant alterations in the T-D- group compared to control animals (T-D+). Resorcin-fuchsin staining density was significantly diminished (Figure 2(a)), suggesting a reduced ratio of noncontractile elements in these vessels. NT immunostaining was significantly elevated (Figure 2(b)), showing an increased formation of nitrogen-derived free radicals in this group.

## 4. Discussion

Our testosterone treatment rat model was appropriate to induce classic PCOS phenotype (hyperandrogenism, ovulatory dysfunction, and PCO morphology of the ovaries). Testosterone levels of the animals following treatment has been published by Hadjadj et al., respectively: T-D+: 0.311 ± 0.16, T + D+: 4.292 ± 0.56, T-D-: 0.720 ± 0.16, T + D-: 5.495 ± 0.56 (ng/ml, mean ± SEM) [10]. Testosterone-treated groups had no estrus cycles [11]. PCO morphology was also a characteristic of testosterone treatment groups, and representative images of the corresponding ovaries were published by Pal et al. [12]. Additional VDD resulted in a more complex disturbance in the carbohydrate metabolism shown by increased HOMA-IR and insulin levels. On the other hand, VDD itself led to a PCOS-like phenotype with modestly elevated testosterone levels, ovulatory dysfunction, and concomitant insulin resistance [10].

Despite achieving PCOS according to standard diagnostic criteria by testosterone treatment in vitamin D supplemented animals, we failed to detect the presumed vascular changes on the level of large vessels. When optimal vitamin D level was provided, hyperandrogenism did not influence either contractile function following NE treatment or relaxation ability after Ach or insulin administration. However, VDD itself seems to have led to marked endothelial dysfunction, in normal as well as hyperandrogenic settings. Similarly, in a previous study, disturbed vasodilation was described in a VDR knockout mouse model [13].

Vitamin D is known to beneficially influence antioxidant systems [14]. In parallel, VDD results in elevated oxidative and nitrative stress. Vitamin D binding to its receptor activates the PI3K/Akt pathway, which is necessary for phosphorylating eNOS in an activating manner. Furthermore, VDR activates the AC/PKA pathway as well as the PIP2/IP3 and PIP2/DAG pathways. These are responsible for elevating intracellular Ca^2+^ levels which is also a permissive factor in eNOS activation. Furthermore, vitamin D is responsible for controlling intracellular redox pathways. By regulating Nrf2/PGC-1alpha-SIRT3 complex, it is essential to maintain normal mitochondrial function. Considering these abovementioned mechanisms, vitamin D deficiency results in elevated oxidative and nitrative stress [15]. In the case of increased production of reactive oxygen species, the spontaneous reaction of superoxide and NO forming peroxynitrite reduces the bioavailability of NO, leaving vasodilation compromised. These nitrogen-derived free radicals can react with protein-tyrosine residues creating 3-nitrotyrosine that can be visualized by NT immunohistochemistry of tissue sections. In our experiments, in concordance with this hypothesis, increased NT staining was observed in the aortic tissue of VDD only (T-D-) animals.

Furthermore, nitrative stress might be a key component in the arising vascular remodeling. In our experiment, VDD only (T-D-) aortas had significantly lower levels of elastic fibers. Nitrative stress results in elevated matrix metalloprotease activity leading to elastinolysis and thus reducing vessel distensibility [16]. This finding also correlates with the results of a previous study where reduced elastic fiber density was found in VDR inactive mutant mice compared to control animals [17]. Another research group induced hypertension with an 84-day long VDD treatment which could be partially explained by impaired vascular structure [18]. On the other hand, it is worth mentioning that vitamin D itself has been widely shown to inhibit elastin production [19].

In our model, testosterone does not act as a definite noxa in terms of vascular function. In our experiment, we saw no changes in vascular function as well as nitrative stress in the T + D+ group. Contrary to assumptions based on previous experiments, testosterone treatment in VDD animals (T + D-) did not worsen but retained the development of endothelial dysfunction and prevented nitrative stress and the deterioration of elastic structures. Furthermore, we witnessed a slight spontaneous elevation in testosterone levels in VDD only (T-D-) group [10], which could be interpreted as a possible compensatory reaction to the damages caused by VDD. Previous studies also suggested that vitamin D can increase aromatase activity and reduce androgen production in various cell lines [20].

Testosterone may have an antioxidant effect as well as vitamin D. This effect has been described primarily in males [21]. Testosterone is also believed to withhold vascular remodeling by reducing matrix metalloprotease levels [22]. Information is lacking about short-term androgen treatment in fertile-age women. The long-term deteriorating effect of supraphysiological androgen levels is proposed in examinations of female-to-male transgender patients [23]. Furthermore, aromatase can convert testosterone to estradiol which has a well-characterized vasodilatory effect in the female aorta [20].

Despite these possible beneficial effects of testosterone, the secondary negative vascular changes caused by metabolic and redox alterations cannot be ignored. These metabolic alterations, including the rising systemic insulin resistance, increased plasma leptin levels, and obesity may impair vascular function in large vessels only in a longer treatment model [10]. A 10-week-long DHT treatment was sufficient to deteriorate ACh-induced vasorelaxation in rat aorta [24]. Besides, the longer treatment period and the different types of androgen used might explain the detected differences in the two models. Vascular changes might arise in sequence from smaller to larger vessels as we detected impaired insulin-induced vasorelaxation in the coronary arteries of our animals following testosterone treatment [10]. Regional differences of vascular insulin resistance were observed compared to coronaries.

Changes in insulin-dependent vasorelaxation cannot be fully explained by the emerging endothelial dysfunction as it robustly affects both VDD groups, differently from ACh relaxation. Nitrative stress and vascular structure remained unchanged in testosterone-treated VDD animals. This suggests a local insulin resistance concordant with the detected systemic insulin resistance in these animals (elevated HOMA-IR in both VDD groups) [10].

## 5. Conclusions

In our study, an 8-week-long testosterone treatment failed to induce vascular dysfunction in the presence of optimal vitamin D supplementation. VDD itself, however, was shown to create a marked endothelial dysfunction accompanied by increased nitrative stress and reduced elastic components of the aortic wall. Short-term testosterone treatment in vitamin D deficiency did not further aggravate the endothelial dysfunction and even prevented nitrative stress and vascular remodeling. Testosterone in PCOS seems to deteriorate large-vessel function in a more chronic, indirect fashion through metabolic and redox disturbances. Further investigation is required to determine the beneficial and detrimental effects of testosterone treatment and identify their time course. In fertile women, reaching optimal serum vitamin D levels is crucial in maintaining large-vessel function as inadequate intake can impair endothelial function and even induce vascular remodeling during a relatively short period of time.

## Data Availability

The data of our research has been uploaded as supplementary material.

## Supplementary Materials

Supplementary Materials Supplementary Description: Norepinephrine T-D+,T + D+,T-DT + D-Acethylcholine T-D+,T + D+,T-DT + D-Insulin T-D+,T + D+,T-DT + D-Vitamin D supplementation. Vitamin D deficiency.
