# Supplementary material for: Effect of Vitamin D Status on Vascular Function of the Aorta in a Rat Model of PCOS
Source: Oxid Med Cell Longev. 2021 Mar 18;2021:8865979. doi: 10.1155/2021/8865979 (PMC7997742; doi:10.1155/2021/8865979)
Supplement: Supplementary Materials — Supplementary Description: Norepinephrine T-D+,T + D+,T-DT + D-Acethylcholine T-D+,T + D+,T-DT + D-Insulin T-D+,T + D+,T-DT + D-Vitamin D supplementation. Vitamin D deficiency. [file 8865979.f1.pdf]

Norepinephrine

T-D+

0,000000001	2,05	1,46	3,73	2,32	1,55	2,5	3,93	3,69	3,14	4,09	1,5
0,00000001	46,68	1,73	17,13	13,54	17,55	12,21	68,73	49,93	32,3	48,35	27,81
0,0000001	88,41	19,39	46,05	53,62	63,2	47,01	105,31	100,7	74,67	104,22	78,19
0,000001	102,57	71,54	53,78	76,67	74,79	51,99	108,32	113,68	90,77	117,7	93,15

T+D+

0,000000001	1,3999	4,138077		1,974426	0,943506	1,037046	2,147511	2,378902	2,218349	2,603735	
0,00000001	16,122	21,11826		22,91017	9,434398	7,441068	10,80213	49,28359	46,58817	33,13754	
0,0000001	38,195	51,84838		66,63312	42,35441	38,27365	39,95555	100,961	98,47553	67,83414	
0,000001	50,36	57,29326		77,42604	55,348	56,55	62,20832	105,4222	107,851	75,99998	

T-D-

0,000000001	11,748	2,2311	3,64228	0,935349	4,315936	4,159046	8,927423	4,290664	2,218359	3,631802	68,52409
0,00000001	64,72	34,64681	53,4548	5,724834	39,16357	46,75808	22,07697	12,50376	14,13543	85,64683	87,59535
0,0000001	107,44	85,53695	95,7371	45,78117	67,73138	82,24044	69,71084	53,93257	51,33629	125,8188	106,1995
0,000001	118,76	95,81761	107,862	62,39953	74,66886	87,62147	82,31056	87,30571	68,30226	130,9118	113,1533

T+D-

0,000000001	0,9263	2,433002	2,90817	12,60804	2,803836	9,007764	4,274011	2,643042	2,537549	5,502835	1,924306
0,00000001	36,051	19,57453	50,5915	65,37479	47,5197	32,23022	36,84947	26,80526	39,22311	42,15329	31,36639
0,0000001	94,039	64,18736	94,1922	104,4479	87,83366	54,61631	85,40273	72,57017	77,18524	75,76622	58,49562
0,000001	109,57	81,61174	102,004	111,8521	98,15797	60,29825	88,65958	69,36006	84,86961	79,7436	60,78066

Acethylcholine

T-D+

0,00000001	6,077662	10,5041	33,66238	28,55866	7,023885	4,43558	10,94827	4,741466	2,1633	6,46564	7,399044
0,0000001	27,93126	74,4192	60,4621	57,76851	43,72097	58,5163	52,78579	38,86709	39,91833	38,1647	51,89775
0,000001	52,67752	92,8411	80,71695	79,18879	69,47298	82,3862	70,04791	58,11267	59,44892	59,9741	74,87692
0,00001	62,83342	95,0137	93,44021	78,70527	73,04596	86,0504	73,66676	60,30987	59,75019	60,2215	79,57315

T+D+

0,00000001	16,91007	29,3381	5,039414	30,51845	11,89473	3,09935	11,76515	13,37427	16,91416
0,0000001	60,13056	79,3437	45,46367	78,27789	52,7556	41,1713	56,26077	50,17835	48,40058
0,000001	83,07041	81,529	79,55375	93,11218	68,76038	70,6471	69,81823	78,06032	73,32739
0,00001	93,7649	95,9646	80,61541	94,80568	66,94091	75,0274	71,55312	82,00001	79,34657

T-D-

0,00000001	1,690172	5,07561	0,560354	10,55858	15,84134	6,71849	4,899737	11,45202	15,79584	26,1339	2,284473
0,0000001	21,39256	10,6655	10,049	24,66855	33,99847	17,3013	32,45667	31,3105	26,02483	37,3494	14,5753
0,000001	48,49381	25,888	29,43152	68,56424	72,41351	45,0574	60,88442	69,24762	54,12332	62,79	31,81655
0,00001	55,21105	34,1181	44,95043	84,074	83,67659	48,94	63,87927	85,52052	58,24117	67,6459	33,57454

T+D-

0,00000001	1,000591	9,1793	6,614483	6,448299	4,89302	34,3868	4,358762	11,84966	9,535407	1,79667	11,07053
0,0000001	1,000591	50,3453	18,65369	45,39919	29,12942	74,1041	28,44016	62,9083	50,46432	21,1982	34,53671
0,000001	17,79635	69,3937	53,58005	62,06414	45,78144	86,4667	60,60899	77,44651	66,92141	54,9411	48,83943
0,00001	26,71293	69,4503	59,90964	62,99719	46,19708	90,5756	54,46274	72,60488	71,09735	53,8682	45,94893

Insulin

T-D+

30	5,163776*	20,70305	22,49085	31,56644	26,49995	38,71764	31,00746	25,32481
120	-9,27082*	37,45252	43,78898	49,6201	42,65384	53,39867	45,54136	41,6705
300	-13,8748*	36,94065	61,4416	60,82725	54,09022	67,20726	57,48351	54,48173
600	-23,5846*	35,44585	62,46941	74,8226	67,24365	75,06681	65,6118	72,75156

T+D+

30	25,25796	50,05081	49,76201	47,37536	36,50656	9,434648	37,41886	21,57306	22,1314	1,731258
120	22,84788	64,06387	73,59828	63,08035	50,15887	19,68206	50,95996	34,24834	32,82592	7,635484
300	25,72169	67,87242	77,82352	69,68426	51,02906	27,96448	62,73811	44,93534	33,54958	17,80262
600	25,25796	74,53221	85,15953	78,3334	36,31379	41,69456	108,6797	58,40604	25,89291	33,59375

T-D-

30	5,249744	16,18509*	13,06804	13,23874	15,66006	4,085376	13,3932	25,67287	4,190165	11,94131
120	7,198683	15,86683*	28,28909	18,60614	21,78496	-0,04735	20,79639	24,79787	5,470262	16,36985
300	6,534658	-2,51852*	19,69334	17,6616	27,31787	-6,55155	27,5151	19,12576	4,709269	1,423507
600	3,806361	-59,2414*	10,42545	1,195217	34,80893	-12,6813	26,51979	3,957998	4,830408	-22,9335

T+D-

30	14,03089	32,4099	22,37596	14,80158	10,77155	18,77622	8,441826	33,62456	17,10379
120	18,73666	32,68718	30,18659	19,68947	9,147618	27,67812	-3,29547	60,85491	22,36301
300	18,1389	24,02943	31,72651	21,63315	4,434597	26,75764	-2,39682	65,84433	20,76381
600	26,2651	18,34094	30,39013	15,48654	-3,5953	46,50622	26,64504	56,09806	-8,81311

NT

	Vitamin D supplementation					
Control	0,112425	0,070951	0,063595	0,045938	0,085642	
Testosterone	0,153989	0,070991	0,11184	0,065262	0,104152	

	Vitamin D deficiency					
Control	0,119715	0,135696	0,085907	0,15082	0,122468	0,119856
Testosterone	*0,046005**	0,113317	0,09963	0,084455	0,08969	0,092996

Resorcin

T- D+	**T+D+**	T- D-	T+ D-
0,380532	0,324673	0,238332	0,353097
0,378942	0,329141	0,240404	0,322175
0,318136	0,328237	0,25421	0,329035
0,368868	0,341742	0,244924	0,316932
0,404422	0,386786	0,227451	0,355764
0,366934	0,354857		
